# The curious consistency of carbon biosignatures over billions of years of Earth-life coevolution

**DOI:** 10.1038/s41396-021-00971-5

**Published:** 2021-04-12

**Authors:** Amanda K. Garcia, Colleen M. Cavanaugh, Betul Kacar

**Affiliations:** 1 Department of Molecular and Cellular Biology, University of Arizona, Tucson, AZ USA; 2 Department of Organismic and Evolutionary Biology, Harvard University, Cambridge, MA USA; 3 Lunar and Planetary Laboratory and Steward Observatory, University of Arizona, Tucson, AZ USA

**Keywords:** Bacterial evolution, Applied microbiology, Biogeochemistry

## Abstract

The oldest and most wide-ranging signal of biological activity (biosignature) on our planet is the carbon isotope composition of organic materials preserved in rocks. These biosignatures preserve the long-term evolution of the microorganism-hosted metabolic machinery responsible for producing deviations in the isotopic compositions of inorganic and organic carbon. Despite billions of years of ecosystem turnover, evolutionary innovation, organismic complexification, and geological events, the organic carbon that is a residuum of the global marine biosphere in the rock record tells an essentially static story. The ~25‰ mean deviation between inorganic and organic ^13^C/^12^C values has remained remarkably unchanged over >3.5 billion years. The bulk of this record is conventionally attributed to early-evolved, RuBisCO-mediated CO_2_ fixation that, in extant oxygenic phototrophs, produces comparable isotopic effects and dominates modern primary production. However, billions of years of environmental transition, for example, in the progressive oxygenation of the Earth’s atmosphere, would be expected to have accompanied shifts in the predominant RuBisCO forms as well as enzyme-level adaptive responses in RuBisCO CO_2_-specificity. These factors would also be expected to result in preserved isotopic signatures deviating from those produced by extant RuBisCO in oxygenic phototrophs. Why does the bulk carbon isotope record not reflect these expected environmental transitions and evolutionary innovations? Here, we discuss this apparent discrepancy and highlight the need for greater quantitative understanding of carbon isotope fractionation behavior in extant metabolic pathways. We propose novel, laboratory-based approaches to reconstructing ancestral states of carbon metabolisms and associated enzymes that can constrain isotopic biosignature production in ancient biological systems. Together, these strategies are crucial for integrating the complementary toolsets of biological and geological sciences and for interpretation of the oldest record of life on Earth.

## Introduction

**Fig. 1:**
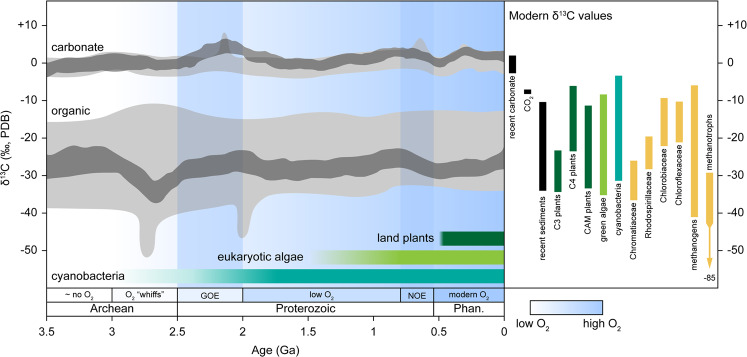
The geologic record of δ^13^C values has remained essentially constant over ~3.5 billion years. Geologic carbonate and organic δ^13^C record (left) and modern δ^13^C values of inorganic carbon and biomass from diverse taxa (right). Light gray fields represent the range of geologic δ^13^C measurements from Schidlowski [6]. Dark gray fields represent 95% confidence intervals for smoothing analyses of geologic δ^13^C data from Krissansen-Totton et al. [7] and references therein. Modern δ^13^C values from Schidlowski [6]. Bars are colored as follows: black, geologic reservoirs; dark green, land plants; light green, green algae; teal, cyanobacteria; other taxa, yellow. Phases of atmospheric oxygen are labeled at the bottom, from Lyons et al. [24] (“~ no O_2_” indicates <10^−5^ present atmospheric level (PAL), “low O_2_” indicates ~10^−1^ to 10^−4^ PAL, and “modern O_2_” indicates PAL). Qualitative O_2_ levels are indicated by shades of blue, with lighter shades indicating lower O_2_ levels and darker shades indicating higher O_2_ levels (also noted by the scale). The Great Oxidation Event (GOE) and Neoproterozoic Oxidation Event (NOE) are indicated by darker blue bars. The earliest potential appearance of cyanobacteria is interpreted from the oldest known oxidized sediments [24, 59, 60]; of eukaryotic algae, from oldest interpreted algal fossils [139] and molecular clock dating [140, 141]; of land plants, from the oldest interpreted pollen fossils [142] and molecular clock dating [143]. PDB Pee Dee Belemnite δ^13^C standard, Phan. Phanerozoic (color figure online).

Life on Earth has generated two main repositories of information with which to reconstruct its past states: first, the genetic diversity of extant organisms, and second, the physical remnants of past life preserved in the geologic record, or biosignatures [1]. By far the most extensive biosignature record—providing the earliest potential evidence of life >3 billion years old [2–5]—is constructed from ^13^C/^12^C isotopic compositions of preserved carbonaceous material, expressed as a normalized value, δ^13^C, typically in units of per mil (‰) [6, 7] (Fig. 1; Box 1).

This carbon isotope record is interpreted to have primarily been shaped by the biological isotopic discrimination of enzymatically driven carbon metabolism. Thus, concerted efforts have been dedicated toward disentangling this record and identifying signals potentially attributable to the metabolic innovations, ecosystem turnover, and global environmental changes that have characterized the history of life [6–10]. Such changes would be expected to manifest in variations to the carbon isotopic record over geologic time. However, deviations in inorganic and organic δ^13^C over the last ~3.5 billion years consistently average ~25‰, creating a largely static trend across the entirety of the record [6, 7]. Only two isolated negative excursions in organic δ^13^C have been resolved, one centered at ~2.7 billion years and the other at ~2 billion years. These excursions have been interpreted to reflect increased methanotrophic and/or methanogenic activity [7, 9, 11, 12]. Why are similar variations not known for other time intervals?

This curious consistency of carbon isotope biosignatures presents an ongoing challenge for interpretation of the most extensive record of life. It is an enormously complex problem, as several factors are known to affect the magnitude of biological isotope discrimination, including diversity in metabolic pathways (Table 1) as well as environmental parameters and host organism physiologies (Table 2). The overlaying of these factors serves to obfuscate individual contributions to preserved carbon biosignatures that might be of interest in understanding the early evolution of life. Further, one must consider the fidelity of the record itself, as the isotopic compositions of poorly preserved geologic samples may be affected by post-depositional abiotic processes that erase primary biogenic signatures [6, 10].

**Table 1:** Isotopic discrimination, productivity, and O_2_ sensitivity of extant carbon fixation pathways.

Pathway	Associated taxa^a^	Modern productivity (Pg C/yr)^b^	^13^C/^12^C isotope discrimination Δδ^13^C (‰)	Isotope discrimination references	Pathway O_2_ sensitive?^b^
CBB	Cyanobacteria [97] Eukaryota [98, 99] photoautotrophs Proteobacteria [100] autotrophic *Alpha-, Beta-, Gammaproteobacteria* Chloroflexi [101] * Oscillochloridaceae* Firmicutes [102] * Sulfobacillus* spp.	~100	~10 to 35	[26, 44, 45, 103–105]	Yes
rTCA	Aquificae [106] * Aquificales* Chlorobi [107] * Chlorobiales* Nitrospirae [108] autotrophs Proteobacteria * Magnetococcus* sp. MC-1 * Desulfobacter hydrogenophilus*	~1	~2 to 23	[44, 45, 103–105]	Yes
roTCA	Aquificae * Thermosulfidibacter takaii* Proteobacteria sulfur-reducing *Deltaproteobacteria*	Unknown	Unknown	N/A	Unknown
HP/HB	Crenarchaeota [109] * Sulfolobales* Marine group I [110] Thaumarchaeota [111]	~0.7	~0 to 4	[44]	No
Wood–Ljungdahl	Euryarchaeota [112] * Archaeoglobales* methanogens Firmicutes acetogens Planctomycetes [113] anaerobic ammonium-oxidizers Proteobacteria autotrophic *Deltaproteobacteria* Spirochaetes [113] * Treponema primitia*	~0.1	~5 to 80	[45, 46, 50, 51, 53, 114]	Yes
DC/HB	Crenarchaeota * Thermoproteales* * Desulfurococcales*	<0.1	~0 to 4	[44, 45]	Yes
3HP	Chloroflexi * Chloroflexaceae*	<0.1	~0 to 14	[20, 44, 115]	No
Reductive glycine^c^	Proteobacteria * Candidatus* Phosphitivorax anaerolimi	<0.1	Unknown	N/A	Unknown

Isotopic discrimination reported from literature, calculated as Δδ^13^C = δ^13^C_product_ − δ^13^C_reactant_, where product is biomass (or acetate/methane for acetogens/methanogens, respectively, utilizing the Wood–Ljungdahl pathway) and reactant is source carbon in growth media.

*CBB* Calvin–Benson–Bassham cycle, *DC/HB* dicarboxylate–4-hydroxybutyrate cycle, *HP/HB* 3-Hydroxyproprionate/4-hydroxybutyrate cycle, *roTCA* reverse oxidative tricarboxylic acid cycle, *rTCA* reductive citric acid cycle, *3HP* 3-hydroxyproprionate bicycle.

^a^Associated taxa from Berg et al. [37], Hugler and Sievert [116], Ward and Shih [25], and references therein. References for definitions of informal taxonomic groups are listed within the table at first instance. Listed taxa are not necessarily diagnostic of each pathway, but rather describe major groups of organisms where pathway can be found.

^b^Modern productivity values from [38, 117–121], and O_2_ sensitivity data from Berg [122].

^c^Recently proposed pathway for the Deltaproteobacterium *Candidatus* Phosphitivorax anaerolimi [36].

**Table 2:** Examples of environmental and physiological factors that affect autotrophic carbon fractionation.

Variable	Taxa	^13^C/^12^C isotope discrimination change (↑ or ↓) with increase in variable	Reference
Temperature	Land plants (varied)	↓ (≤ ~4‰)	[123]
	Diatoms (varied)	↓ (≤~7‰; less change at high [CO_2_])	[124]
	Marine plankton (varied)	↑ or ↓, dependent on taxa (≤ ~4‰)	[125]
pH	*Spinacea oleracea* (land plant)	↓ (≤ ~3‰)	[15]
	*Skeletonema costatum* (diatom), *Emiliania huxleyi* (coccolithophore)	↑ or ↓, dependent on pH range (≤ ~9‰)	[47]
CO_2_ concentration	Land plants (varied)	↑ (≤ ~7‰)	[126]
	*Skeletonema costatum* (diatom), *Emiliania huxleyi* (coccolithophore)	↑ (≤ ~7‰)	[47]
	* Emiliania huxleyi* (coccolithophore)	↑ (≤ ~7‰)	[127]
	*Emiliania huxleyi* (coccolithophore)	↑ (≤ ~7‰)	[128]
Growth rate	*Phaeodactylum tricornutum* (diatom)	↓ (≤ ~20‰)	[129]
	*Emiliania huxleyi* (coccolithophore)	↓ (≤ ~7‰)	[128]
	Marine plankton (field samples)	↓ (≤ ~9‰)	[130]
	Marine plankton (field samples)	↓ (≤ ~8‰)	[131]
Cell surface area:volume	Marine plankton (varied)	↑ (≤ ~20‰)	[132]
H_2_ concentration	*Methanothermobacter marburgensis* (methanogen)	↓ (≤ ~30‰)	[133]
	*Methanocaldococcus jannaschii* (methanogen)	↓ (≤ ~16‰)	[134]
Pressure	*Methanopyrus kandleri* (methanogen)	↓ (≤ ~22‰)	[135]

Here, in addition to surveying several biological and environmental factors that quantitatively affect carbon biosignatures, we contend with an important aspect that is not typically considered—the potential role of subcellular evolution in shaping the carbon isotope record. We discuss in particular the evolution of the CO_2_-fixing enzyme RuBisCO (ribulose 1,5-bisphosphate carboxylase/oxygenase, EC 4.1.1.39) [13, 14], which produces comparable isotope effects in extant oxygenic phototrophs (ε ~20 to 30‰; see Box 1 for a discussion of isotope effects) to the ~25‰ mean isotopic difference between preserved inorganic and organic carbon [15–19]. RuBisCO is the catalytic bottleneck of the Calvin–Benson–Bassham (CBB) cycle used primarily by oxygenic phototrophs, though also by certain Proteobacteria, Gram-positive bacteria, and Chloroflexi [20–22] (Table 1). The CBB cycle facilitated by oxygenic phototrophs evolved early in Earth history, at least by 2.4 billion years as evidenced by broadly accepted geochemical signatures of atmospheric oxygen [23, 24]. This, in addition to the predominance of oxygenic phototrophy in modern primary production, suggests that RuBisCO has been the most important driver of carbon fixation for much of Earth history [6, 25].

There are several reasons to expect that molecular-level changes to RuBisCO enzymes over geologic history may have been imprinted upon the carbon isotope record. The range of isotope effects for differing forms of RuBisCO can extend outside that associated with well-studied oxygenic phototrophs (i.e., ε ~< 20‰; Table 3). Furthermore, extant RuBisCO carbon uptake efficiency varies as a function of external CO_2_ levels and protein sequence variation, which subsequently affects the degree of carbon isotope fractionation [16, 19, 26–28]. Because atmospheric CO_2_ levels have changed markedly over Earth history [29], one would expect molecular adaptations in RuBisCO CO_2_ specificity to thus be expressed in carbon biosignatures.

**Table 3:** Available measurements of ^13^C/^12^C isotope effects (ε) from diverse forms of purified RuBisCO enzyme, measured under saturating CO_2_ levels.

Group	Species	RuBisCO form	^13^C/^12^C isotope effect, ε (‰)	Isotope effect reference
Proteobacteria	*Solemya velum* bivalve symbiont	IA	24.4	[19]
Cyanobacteria^a^	*Prochlorococcus marinus* MIT9313	IA	24.0	[16]
Cyanobacteria^a^	*Synechococcus elongatus* PCC6301	IB	22.0	[17]
Land plant	*Flaveria bidentis*	IB	27.8	[18]
Land plant	*Flaveria floridana*	IB	28.6	[18]
Land plant	*Nicotiana tabacum*	IB	29	[18]
Land plant	*Spinacia oleracea*	IB	28.2–30.3	[15, 17, 19]
Proteobacteria	*Ralstonia eutropha*	IC	19.0	[136]
Proteobacteria	*Rhodobacter sphaeroides*	IC	22.4	[136]
Coccolithophore	*Emiliana huxleyi*	ID	11.1	[137]
Diatom	*Skeletonema costatum*	ID	18.5	[27]
Proteobacteria	*Rhodospirillum rubrum*	II	17.8–23.8	[17, 18, 26, 73]
Proteobacteria	*Riftia pachyptila* symbiont	II	19.5	[74]

^a^Horizontal transfer of RuBisCO Form I genes likely occurred within cyanobacteria [72, 138].

An advantage of this molecular perspective is that the expectations for ancient variation in RuBisCO isotopic fractionation can be experimentally tested. Recently, molecular paleobiology has been recruited to reconcile independent biological and geological records of life by the laboratory reconstruction of ancestral enzymes and metabolic systems responsible for producing preserved biosignatures [30–32]. A fundamental issue with the interpretation of carbon isotope biosignatures is that it is not known to what extent the isotope discrimination behavior or modern biology can serve as a proxy for past life. These paleogenetic tools instead leverage modern genomic information and phylogenetic models to infer the molecular sequences of ancestral enzymes prior to their experimental synthesis and characterization [32, 33]. By this approach, the isotopic effects of inferred ancestral enzymes can be compared directly with preserved carbon isotope biosignatures, thereby reconciling biological and geological records of life [30]. Such an approach is not itself meant to be a complete solution to understanding the consistency of the carbon isotope record. Rather, these strategies can help constrain the set of contributing factors and complement further characterization of extant biological fractionation processes and the geological samples themselves. Together, these efforts provide an empirical strategy to interrogate the oldest physical remnants of ancient life.

Box 1. Mechanism and measurement of biological carbon isotope discrimination Measurements of carbon isotope compositions are relative, normalized to an international standard (Vienna Pee Dee Belemnite), and expressed as:

$$\delta ^{13}{\rm{C}} = \left[ {\left( {{\,}^{13}{\rm{C}}_{\rm{sample}}/{\,}^{12}{\rm{C}}_{\rm{sample}}} \right)/\left( {{\,}^{13}{\rm{C}}_{\rm{standard}}/{\,}^{12}{\rm{C}}_{\rm{standard}}} \right) - 1} \right]$$

Materials enriched in ^13^C relative to the standard (defined as δ^13^C = 0‰) are thus expressed as positive δ^13^C values, whereas those depleted in ^13^C, like biogenic carbon compounds, are expressed as negative δ^13^C values.

In autotrophic organisms, CO_2_ fixing enzymes typically discriminate against the heavier ^13^C isotope as a result of an enzymatic kinetic isotope effect that leaves the resulting product fractionated, or depleted, in ^13^C relative to the CO_2_ source. The heavier ^13^C isotope requires greater activation energy to reach the transitional state, resulting in a slower reaction rate. The degree to which a given carboxylase discriminates is measured as a ratio of the reaction rate constants for each isotope (k^12^:k^13^), which is converted to an epsilon value, ε = 1000 [(k^l2^/k^13^) − 1]. For example, for RubisCO, this is directly proportional to the discrimination by the enzyme against ^13^C that occurs between the substrate pool (CO_2_) and the product (phosphoglycerate; [144]).

Enzyme fractionation has been approximated in whole cells in laboratory experiments where the δ^13^C of the source CO_2_ in the growth media is known (Table 1). This is calculated as Δδ^13^C = δ^13^C_biomass_ − δ^13^C_CO2_. Since Δδ^13^C values encompass other physiological factors also influencing carbon isotope ratios of samples (see Table 2), ε as defined above is restricted here for carbon fractionation measured for purified enzymes.

## The production and preservation of carbon isotopic biosignatures

Several biotic and abiotic factors are known to influence the magnitude of isotopic fractionation as carbon is assimilated into biomass. At the heart of carbon fixation pathways, enzyme fractionation associated with the production of biological carbon is the result of an enzymatic kinetic isotope effect that produces differences in the δ^13^C compositions of substrates versus products (see Box 1 for a discussion on fractionation mechanism as well as a description of notation used here). These effects arise from the isotope mass difference between ^13^C and ^12^C [34, 35], and result in a slight preference for the conversion of ^12^C-containing compounds to organic biomass. In addition to this enzymatic effect, environmental and physiological factors can additionally modulate the isotopic composition of fixed carbon.

There are seven known pathways of carbon fixation utilized by autotrophs, including the aforementioned CBB cycle and the recently proposed reductive glycine pathway [36] (reviewed in [25, 37]) (Table 1). These different autotrophic pathways vary in their taxonomic distributions, oxygen sensitivities, and contributions to total modern primary productivity. Though the CBB pathway is today responsible for the bulk of total fixed carbon [38–40], it is not considered to be the oldest carbon fixation mechanism. Instead, it has been suggested that the Wood–Ljungdahl pathway, today utilized by acetogenic and anaerobic ammonium oxidizing bacteria as well as methanogenic archaea, is the oldest mechanism and is proposed to be associated with the last universal common ancestor [25, 41–43].

The Δδ^13^C values associated with different carbon fixation pathways are calculated as the difference between δ^13^C of biomass and source dissolved inorganic carbon or CO_2_ (and thus reflect a combination of enzymatic, physiological, and environmental effects). Δδ^13^C measured for the rTCA, HP/HB, DC/HB, and 3HP pathways indicate smaller isotopic discriminations (Δδ^13^C = 0–15‰) relative to those produced by the CBB pathway (Δδ^13^C = 10–30‰) (Table 1). The Wood–Ljungdahl pathway can produce discrimination in excess of that of the CBB pathway (i.e., Δδ^13^C = 30–40‰) [44, 45], with the greatest discrimination (Δδ^13^C = 65‰) measured from acetogens [46]. Δδ^13^C values have not yet been determined for the roTCA and recently described reductive glycine pathways.

Environmental and physiological components of Δδ^13^C values have themselves also been investigated (Table 2). Changes in physical environmental factors, including temperature, pH, and CO_2_ and H_2_ concentrations (the latter in methanogenic organisms) all have significant effects, typically imparting between a 5 and 30‰ change in isotope discrimination. Decreased external CO_2_ concentrations in particular appear to reliably result in decreased isotopic fractionations. This is likely due to a Rayleigh distillation effect in which, at low CO_2_ concentrations, organisms will use intracellular CO_2_ faster than can be diffusively exchanged with external source CO_2_, thereby minimizing isotopic discrimination as well [47]. This relationship has been leveraged in an effort to use carbon isotopic compositions of preserved organic matter as a proxy for ancient atmospheric CO_2_ levels [8, 48, 49]. For variables that have been tested using methanogen cultures, including H_2_ concentration and pressure, fractionation can vary up to 30‰. Physiological factors, including growth rate and cell shape, can result in up to ~20‰ variability in isotope discrimination. In sum, these environmental and physiological factors can produce variability in fractionation that can meet or exceed variability attributed to differences in autotrophic metabolic pathways. However, the majority of these studies have been conducted on organisms such as eukaryotes that likely only evolved after the first 1–2 billion years of Earth history (Table 2). The autotrophic organisms more likely to have contributed to the first half of the geologic carbon isotope record are significantly underrepresented in studies of carbon isotopic fractionation.

The fate of fixed carbon is further biological recycling and/or burial. The former can result in significant carbon isotopic fractionation effects that can overprint autotrophic signatures. For example, methylotrophic methanogens (i.e., utilizing single carbon substrates other than CO_2_) can produce Δδ^13^C values as great as ~80‰ between product CH_4_ and source inorganic carbon [50, 51]. Further, methanotrophy, itself isotopically discriminating by ~30‰ [52], can result in an even greater depletion of ^13^C in biomass given an initially ^13^C-depleted CH_4_ substrate (δ^13^C = ~60‰) produced by methanogens. Abiotic, post-depositional processes can further alter primary biogenic isotope signatures. Thermal alteration associated with metamorphism, for example, results in preferential loss of ^12^C from preserved organic material [10]. Thus, care must be taken in interpreting carbon biosignatures from potentially altered samples. Independent methods for assessing thermal alteration, such as H/C content, can be used to quantify the degree of thermal maturation in filtering the carbon biosignature dataset [10].

In sum, the variability between isotope fractionation among different carbon metabolisms permits isotope compositions to not only be used broadly as a biosignature of life but can generally be used to fingerprint particular metabolic processes and the taxa associated with them. The portion of carbon that is subsequently preserved in the geologic record thus forms a remnant signature of these ancient carbon cycling processes.

## Features of the carbon isotope record

The isotopic compositions of organic and inorganic carbon preserved in the geologic record, spanning >3.8 billion years, together provide the oldest forms of preserved ancient biosignatures [6, 7] (Fig. 1). Scales of isotopic measurement can range from bulk rock characterizations to those of individual, microscopic, organic fossils (e.g. [5, 53–55],). The absolute difference between inorganic and organic δ^13^C values in the geologic record is interpreted to reflect contemporaneous, biological isotopic fractionating processes including carbon fixation [6, 7, 10].

To date, the purportedly oldest biogenic carbon isotope measurements have been obtained from submicron graphitic inclusions in a ~4.1-billion-year-old zircon. These measurements yield a δ^13^C value of −24 ± 5‰, falling within the range of biological fractionation (Fig. 1) providing the earliest potential evidence of life [2]. The biogenicity of such ancient measurements is subject to controversy, owing primarily to the potential for more recent alteration of these isotopic signatures and the influence of comparable abiotic fractionation processes on the early Earth. Unambiguous assignment of these isotopic values to any particular metabolic process has not yet been achieved. For the more recent <3.5 billion years of the carbon isotope record, biogenicity of such signatures is less contentious due to a nearly coincident morphological fossil record [56, 57]. Furthermore, organic matter in more recent sediments is typically found as amorphous kerogen rather than graphite, the latter of which is more likely to have been produced abiotically and/or potentially indicative of high thermal alteration [10, 58].

Efforts have been made by geochemists and paleobiologists to filter the carbon isotope dataset to minimize representation of samples more likely to have been affected by post-depositional alteration, as well as statistically evaluate trends in the record [7]. After such treatments, a largely static isotopic trend remains but is punctuated by a significant negative excursion in organic δ^13^C coinciding with the late Archean to Proterozoic transition, previously noted by Hayes [9]. This excursion has been interpreted to represent the increased activity of oxygen-requiring methanotrophy. Methanotrophic recycling of buried organic material may have accompanied the initial accumulation of free oxygen following the evolution of oxygenic phototrophs. Since methanotrophy can result in exceedingly ^13^C-depleted carbon as described above [52], the influence of this metabolic process is a reasonable explanation for the identified excursion. Another negative isotopic anomaly, though not identified by Krissansen-Totton et al. [7], has been noted at ~2 billion years [12]. This excursion may have similarly resulted from the contributions of methanotrophs or methanogens that both produce large isotopic discriminations in excess of that typically observed for oxygenic phototrophs. On more recent geologic timescales, finer trends in the last 100 million years have been attributed to changes in atmospheric CO_2_ concentrations due to the empirical relationship between CO_2_ concentration and isotopic discrimination, as described above [8, 48, 49].

## A molecular perspective on the role of rubisco evolution in shaping the carbon isotope record

Though compelling, the few identifiable signals serve to heighten the curious consistency in the remainder of the carbon isotope record, particularly considering the dynamic, early Earth biogeochemical environment. One of the most fundamental shifts in the biosphere over Earth’s 4.5-billion-year history has been the progressive oxygenation of the surface environment, mediated by the origin of oxygenic photosynthesis [24]. This process may have begun with early “whiffs” of oxygen by ~3 to 2.5 billion years ago [59, 60], but unambiguous signatures of atmospheric oxygen are not known before 2.4 billion years ago [23]. The isotopic excursion in the late Archean-early Proterozoic is likely related to these changes in environmental oxygen. However, it is still unclear why other deviations are not present due to the expected breadth of biological consequences from such a significant shift in atmospheric composition [57, 61, 62]. Furthermore, oxygen levels likely remained exceedingly low through the Proterozoic until ~0.5 billion years ago [24], yet biological isotopic trends associated with later shifts in atmospheric composition are not readily identifiable.

The first 3 billion years—the vast majority—of the carbon isotope record, produced primarily by the ancient microbial organisms that dominated the Precambrian Era (~0.54 to 4.5 billion years ago), likely requires a different level of analysis than that of the more recent geologic past. In the absence of the later-evolved, multicellular eukaryotes that typify the Phanerozoic Era (present to ~0.54 billion years ago), the early evolution of life and the major biological innovations that occurred through the Precambrian have frequently been considered rather at the subcellular level. For example, focus has been drawn to the molecular machines, enzymes, that catalyze crucial biogeochemical transformations and shaped primary productivity for billions of years [30, 39, 63, 64]. Comparatively, little attention has been given to how the molecular evolution of carbon fixation enzymes may have impacted and/or constrained features of the carbon isotope record.

This perspective can be illustrated in the evolution of the early-evolved RuBisCO enzyme, which plays a critical role in the modern biosphere and is proposed to be one of the most abundant proteins on Earth [65–67]. This enzyme is thought to have evolved more than 3 billion years ago in the anoxic environment preceding the Great Oxidation Event [13, 14, 68, 69]. Today, three forms (I–III) of RuBisCO catalyze carbon uptake in the CBB cycle; a fourth form (IV), a “RuBisCO-like” enzyme, is homologous but does not perform a carboxylase function [21, 70]. Cyanobacteria, green algae, and land plants utilize Form IA and IB RuBisCO, suggesting that the evolution of these forms are linked to that of Earth’s dominant phototrophs [71]. However, previous phylogenetic analyses indicate that other forms of RuBisCO diverged earlier than Form IA and IB homologs [13, 72]. It is then possible that ancestral enzymes preceding the evolution of oxygenic phototrophy shared greater similarity to the catalytic properties, and thus, isotopic effects, of earlier diverged forms.

However, isotopic fractionation measurements of purified RuBisCO enzymes are few, even for the better-characterized Form IA and IB enzymes. Isotope effects have been measured for certain Form IC and ID enzymes associated with coccolithophores, diatoms, and proteobacteria (ε ~11 to 22‰), which are generally distinguishable from effects for Form IA and IB (ε ~20 to 30‰) (Table 3). Isotope effects from only two organisms have been measured for Form II RuBisCO [17, 18, 26, 73, 74], and no fractionation values have been measured from Form III, which phylogenetic analysis indicates diverged earlier than Form I and II RuBisCO homologs [72]. Thus, characterization of Form III, as well as other underrepresented forms (e.g., Form IC, ID, and II), are necessary to evaluate their potential impact on the early carbon isotope record.

An anaerobic origin of RuBisCO is also of interest in the context of its substrate specificity. In addition to CO_2_ assimilation, RuBisCO catalyzes a competing oxygenation reaction in which RuBP is combined with O_2_, which in turn reduces the overall metabolic efficiency of carbon fixation. RuBisCO specificity inversely correlates with enzyme activity [75–78]. It has been argued that the balance between specificity and enzyme activity is achieved by the RuBisCO transition state, which accentuates the structural differences between otherwise similar CO_2_ and O_2_ molecules at the cost of slowed catalysis [77]. For an organism that makes its living by RuBisCO-catalyzed CO_2_ fixation, an atmosphere with significant amounts of O_2_—as well as cellular O_2_ produced by oxygenic photosynthesis—presents a serious hindrance [79, 80]. Extant organisms compensate for this inefficiency by various strategies, including active CO_2_-concentration mechanisms [81, 82]. However, such strategies would have been unnecessary during the early evolution of RuBisCO, prior to the evolution of oxygenic phototrophs when atmospheric CO_2_ concentrations may have been up to 2500 times higher than today [24, 28, 29, 83]. This suggests that the O_2_/CO_2_-specificity problem may only be significant in the O_2_-rich atmosphere that has characterized the latter half of Earth’s history [24].

Analyses of extant RuBisCO isotope effects show that changes in O_2_/CO_2_-specificity and catalytic efficiency manifest in changes to isotope effects [16, 19, 26, 27, 77]. A study of more than 100 diverse Form II and III RuBisCOs only recently found that the range of carboxylation rates extends to more than twofold that of plant RuBisCOs [84]. This may suggest that the true diversity of RuBisCO fractionation behavior is similarly not captured by existing measurements. If the specificity of RuBisCO adapted because of secular trends in environmental O_2_/CO_2_ levels, these changes would be expected to manifest in the carbon isotope record.

## Future directions—paleogenetic reconstruction of ancestral carbon fixation pathways

There are several reasons why molecular-level adaptations to RuBisCO (as well as other carbon fixation enzymes) to the changing early Earth environment would leave discernible features in the carbon isotope record, as described above. These expectations warrant empirical testing. However, the use of extant biology as a proxy for ancient life is fundamentally limited. It is not known to what degree isotopic fractionation of modern organisms and their subcellular components resemble that for the enzymes, metabolic networks, and organisms that existed billions of years ago. For instance, the landscape of early carbon metabolic networks, which would have manifested from predecessor prebiotic chemical networks [85], may have been fundamentally different during and immediately after the origin of life [86, 87]. Thus, isotopic fractionation expectations derived from features of modern biology may be inherently limited in their scope.

We propose that this challenge can be met by combining the complementary strengths of geological and biological datasets. This can be accomplished through the integration of molecular paleobiology and synthetic biology tools to reconcile ancestral enzyme behaviors with the geochemical record of biological activity. This strategy applies phylogenetic models to extant genomic data to reconstruct the molecular sequences of ancestral enzymes [32, 33]. These sequences can then be synthesized in the laboratory and experimentally characterized for properties of interest. Though inferred sequences are probabilistic, they can serve to constrain the molecular sequence space that results in a particular phenotype. In vitro assessments of enzymatic properties can be conducted in addition to in vivo studies that consider the impact of ancestral enzymes on the physiology of the host organism [30, 88]. Such an approach has had prior success in elucidating fundamental features of molecular evolution. These include the evolution of enzymatic specificity [89], origins of novel functions [90, 91], and ancient enzyme promiscuity [92, 93]. However, its application to biogeochemical questions, in particular those related to the generation of ancient biosignatures, is in its infancy [30].

This strategy, for example, could be leveraged to experimentally test the relationship between CO_2_ specificities and isotopic effect in ancestral RuBisCO. This work could confirm expectations for deviations in carbon isotope biosignatures due to observed fractionation effects that do not conform to that observed in the geologic record. Thus, the consistency of the record would then require explanation by other factors that would balance this deviation. Alternatively, ancestral RuBisCOs might fractionate carbon much like their extant counterparts. This result would support the possibility that the molecular evolution of RuBisCO has been fundamentally constrained with regard to isotopic fractionation behavior despite long-term adaptations to the Earth’s atmosphere. In vivo experiments as described above can help determine to what extent physiological properties can overprint enzyme-level isotopic effects.

A comprehensive approach to molecular paleobiology could in the future be expanded to other carbon fixation enzymes in, for example, anaerobic taxa that might have been predominant prior to the origin of oxygenic phototrophy. Other analyses might incorporate compound-specific or site-specific isotopic investigations to work in concert with their increased use as geochemical proxies [94, 95]. These strategies would thus bridge molecular, organism, and environmental factors in disentangling the contributions to carbon isotope biosignatures. At an even broader level, molecular paleobiology techniques may also be applied to reconcile other enzyme-implicated signals in sulfur or nitrogen isotopic systems [96] or to investigate a more expansive array of ancient organic molecular biosignatures that changed over macroevolutionary timescales.

The appeal of developing new paleogenetic techniques is found in the integration of biological and geological records of life, and the recruitment of molecular biology communities toward longstanding challenges in ancient microbial ecology and biogeochemistry. We propose that this strategy, molecular paleobiology approaches used in concert with expanding microbiological and geochemical toolsets to characterize extant taxa and refine the carbon isotope dataset, will rapidly advance resolving the long-term conundrum of carbon biosignatures observed in the fossil record. What is at stake is the fundamental notion of the pervasiveness and universality of carbon isotope biosignatures, particularly as such analyses extend beyond Earth. Future work will expose the extent to which paleobiologists, microbiologists, geochemists, and planetary scientists understand the carbon isotope record, one of the foundational interpretive tools to reconstruct past biological activity.
